# High-pressure versus low-pressure home non-invasive positive pressure ventilation with built-in software in patients with stable hypercapnic COPD: a pilot study

**DOI:** 10.1038/s41598-017-17142-2

**Published:** 2017-12-01

**Authors:** Luqian Zhou, Lili Guan, Weiliang Wu, Xiaoying Li, Xin Chen, Bingpeng Guo, Yating Huo, Jiawen Xu, Yuqiong Yang, Rongchang Chen

**Affiliations:** 1 State Key Laboratory of Respiratory Disease, Guangzhou Institute of Respiratory Disease, the First Affiliated Hospital of Guangzhou Medical University, Guangzhou, China; 2 The First Affiliated Hospital/School of Clinical Medicine of Guangdong Pharmaceutical University, Guangzhou, China; 3 ZhuJiang Hospital of Southern Medical University, Guangzhou, China

## Abstract

High-pressure non-invasive positive pressure ventilation (NPPV) is a new strategy targeted at maximally reducing arterial carbon dioxide. However, high inspiratory positive airway pressure (IPAP) might cause respiratory adverse events likely to diminish the benefit of NPPV. In the setting of ventilatory support, monitoring NPPV efficacy and resolving problems promptly are critical. This study assessed the treatment effect of high and low-pressure NPPV in chronic hypercapnic COPD using home ventilator with built-in software. In this pilot study, we investigated 34 patients using NPPV for 3 months. 13 patients used high-pressure ventilation and 21 patients used low-pressure ventilation. The primary outcome was daytime partial pressure of arterial blood carbon dioxide (P_a_CO_2_). There were no between-group differences in daytime P_a_CO_2_ and FEV_1_, but a trend favouring high-pressure NPPV was observed. Significant between-group differences were found in the transition dyspnoea index (TDI) (high-pressure, 1.69 ± 1.75, versus low-pressure, −0.04 ± 2.71, *p* = 0.044). No differences were found in usage time, leakage, health-related quality of life, spirometry, or 6-minute walk test. High-pressure NPPV with built-in software monitoring in patients with chronic hypercapnic COPD is associated with improvement in TDI scores and a positive trend in favour of high-pressure NPPV for improving P_a_CO_2_ is observed.

## Introduction

Chronic obstructive pulmonary disease (COPD) is a chronic airway disease characterized by incompletely reversible airflow limitation. Currently, COPD is the fourth major cause of death in the world. Advanced-stage COPD is the most common cause of dyspnoea and respiratory failure, with hypoxemia or hypercapnia resulting from respiratory muscle fatigue and alveolar hypoventilation. Non-invasive positive pressure ventilation (NPPV) is considered for patients with chronic hypercapnic COPD (Evidence B)^1^. NPPV can help patients reduce the respiratory muscle workload and increase the alveolar ventilation volume, thus rectifying hypercapnia, improving oxygenation, and relieving shortness of breath^1,2^. However, different investigators have drawn various—even opposing—conclusions^3–8^. The reasons for these disputed results are unclear, perhaps they result from the lower inspiratory pressures that had been used in some randomized controlled trials.

High-intensity NPPV refers to particular NPPV settings that are using assist/control mode aimed at maximally improving the partial pressure of arterial blood carbon dioxide (P_a_CO_2_), with inspiratory positive airway pressure (IPAP) 20–30 cm H_2_O^9–14^. Compared with low-intensity NPPV, high-intensity NPPV could significantly reduce P_a_CO_2_ and even improve pulmonary function^11^. Results from one study showed that high-intensity NPPV was able to better improve the gas exchange and reduce inspiratory effort, and it led to nearly a complete rest of the diaphragm^15^. Some authors have pointed out that high inspiratory pressure played a significant role in high-intensity NPPV and there was no additional benefit adding a high back-up rate to high-pressure NPPV^16^. So far, no definitive conclusion reached about whether high-pressure NPPV is the best approach for the long-term treatment of patients with hypercapnic COPD. In addition, when using high-pressure NPPV, high IPAP might lead to excessive leakage, patient-ventilator asynchrony, and other respiratory adverse events, as well as adverse effects on cardiac performance. These factors probably reduce patient compliance with treatment^11,15,17,18^. Hence, monitoring the efficacy of home NPPV and fixing problems promptly are of great significance.

The objective of this pilot study was to test the feasibility and compare the efficacy of high-pressure NPPV with that of low-pressure NPPV in patients with chronic hypercapnic COPD using a non-invasive home ventilator equipped with built-in software.

## Results

A total of 34 patients were included in this study, 13 patients in the high-pressure group and 21 patients in the low-pressure group. Baseline demographics were similar in both treatment groups (Table 1). The mean IPAPs in the high-pressure and low-pressure groups were 21.15 ± 1.34 cm H_2_O and 14.93 ± 0.87 cm H_2_O, respectively. Treatment compliance was good in both groups. Non-invasive ventilator (NIV) use time in both groups was similar (high-pressure group, 362.41 ± 99.69 minutes versus low-pressure group, 343.55 ± 74.23 minutes; *p* = 0.538). More leakage was detected in the high-pressure group; however, no significant difference was discovered between groups (high-pressure group, 40.57 ± 12.52 L/min versus low-pressure group, 37.11 ± 11.95 L/min; *p* = 0.169).

**Table 1:** Baseline demographic and clinical characteristics.

	High-pressure ventilation	Low-pressure ventilation
Male, n (%)	11 (84.62)	14 (66.67)
age, years	70.38 ± 6.13	67.81 ± 6.99
BMI, kg/m_2_	19.13 ± 2.61	20.76 ± 3.76
SaO_2_, %	89.08 ± 6.14	91.90 ± 4.28
IPAP, cmH2O	21.15 ± 1.34	14.93 ± 0.87
EPAP, cmH2O	4.31 ± 0.48	4.38 ± 0.59
pH	7.37 ± 0.02	7.38 ± 0.03
PaCO_2_, mmHg	58.16 ± 6.48	58.85 ± 7.48
PaO_2_, mmHg	68.89 ± 15.40	65.60 ± 13.99
FVC, L	1.55 ± 0.64	1.49 ± 0.46
FVC, % predicted	48.18 ± 18.63	47.59 ± 17.92
FEV_1_, L	0.55 ± 0.11	0.53 ± 0.16
FEV_1_, % predicted	22.10 ± 5.29	24.66 ± 9.58
FEV_1_/FVC, %	39.27 ± 13.85	37.31 ± 10.61
BDI	4.31 ± 1.60	5.10 ± 2.07
Usage time, min	362.41 ± 99.69	343.55 ± 74.23
Leakage, L/min	40.57 ± 12.52	37.11 ± 11.95

Values represent as means ± SD.; BMI, body mass index; SaO_2_, arterial oxygen saturation; IPAP, inspiratory positive airway pressure; EPAP, expiratory positive airway pressure; FEV_1_, forced expiratory volume in 1 s; FVC, forced vital capacity; PaCO_2_, arterial carbon dioxide pressure; PaO_2_, arterial oxygen pressure; BDI, Baseline Dyspnea Index.

For the primary outcome, no significant between-group difference could be found in daytime P_a_CO_2_ (high-pressure group, 47.40 ± 5.23 mmHg versus low-pressure group, 51.67 ± 7.40 mmHg, *p* = 0.058). A positive trend in the difference between both groups was noted and the same trend was seen in FEV_1_ (high-pressure group, 0.62 ± 0.11 L versus low-pressure group, 0.55 ± 0.21 L, *p* = 0.065). Moreover, the transition dyspnoea index (TDI) of the high-pressure group was improved, and a significant difference between both groups was observed (high-pressure group, 1.69 ± 1.75 versus low-pressure group, −0.04 ± 2.71, *p* = 0.044). In addition, the health-related quality of life (HRQL) (Severe Respiratory Insufficiency (SRI) and the COPD assessment test (CAT)) improved in both groups; however, no between-group differences were seen (SRI: high-pressure group, 54.43 ± 13.74 versus low-pressure group, 52.95 ± 10.28, *p* = 0.722; CAT: high-pressure group, 21.77 ± 5.92 versus low-pressure group, 22.24 ± 6.67, *p* = 0.837). (Table 2) There were no differences in arterial oxygen saturation, FVC, pH, or partial pressure of arterial blood oxygen (P_a_O_2_) between the high-pressure and low-pressure groups. Furthermore, the percentage of the changes in the 6-minute walk distance (6MWD) reached minimal clinically important difference of 30 m was similar in both groups: seven patients in the high-pressure group (53.85%) versus eight patients in the low-pressure group, (38.10%); *p* = 0.484.

**Table 2:** Comparison of baseline and treatment for High-pressure NPPV and low-pressure NPPV.

	High-pressure ventilation		Low-pressure ventilation		p
	Baseline	3 months	Baseline	3 months	
SaO_2_	89.08 ± 6.14	91.85 ± 6.15	91.90 ± 4.28	91.10 ± 5.04	0.43
FEV_1_	0.55 ± 0.11	0.62 ± 0.11	0.53 ± 0.16	0.55 ± 0.21	0.07
FVC	1.55 ± 0.64	1.70 ± 0.68	1.49 ± 0.46	1.58 ± 0.50	0.59
pH	7.37 ± 0.02	7.40 ± 0.03	7.38 ± 0.03	7.39 ± 0.03	0.26
PaCO_2_	58.16 ± 6.46	47.40 ± 5.23	58.85 ± 7.48	51.67 ± 7.40	0.06
PaO_2_	68.89 ± 15.40	73.14 ± 12.56	65.60 ± 13.99	68.42 ± 17.20	0.27
SRI	45.36 ± 11.95	54.43 ± 13.74	44.98 ± 8.82	52.95 ± 10.28	0.72
CAT	25.77 ± 3.75	21.77 ± 5.92	26.43 ± 5.06	22.24 ± 6.67	0.84
TDI	—	1.69 ± 1.75	—	−0.04 ± 2.71	0.04

Values represent as means ± SD.; NPPV, non-invasive positive pressure ventilation; SaO_2_, arterial oxygen saturation; FEV_1_, forced expiratory volume in 1 s; FVC, forced vital capacity; PaCO_2_, arterial carbon dioxide pressure; PaO_2_, arterial oxygen pressure; SRI, Severe Respiratory Insufficiency; CAT, COPD assessment test; TDI, Transitional Dyspnoea Index.

## Discussion

To the best of our knowledge, this is the first pilot study comparing the treatment effect between high-pressure NPPV and low-pressure NPPV in chronic hypercapnic COPD patients using a non-invasive home ventilator equipped with built-in software in Asia. The software allowed early identification and prompt resolution of adverse events during use. In this study, the association between high-pressure NPPV and the improvement in TDI scores was found when compared with low-pressure NPPV, however, no significant between-group differences were detected in leakage, compliance, daytime P_a_CO_2_, pulmonary function, HRQL, and exercise tolerance.

Interestingly, a between-group difference was observed in TDI. Also, FEV_1_ was shown to have a positive trend in improvement, probably because of airway dilatation or the anti-inflammatory effect of using NPPV^19,20^. Another essential reason might be the mitigation of airway oedema. Carbon dioxide retention can lead to vasodilation, which may result in oedema. Decreasing P_a_CO_2_ level after using home NPPV, this is especially true for high-pressure NPPV, which could restore daytime P_a_CO_2_ to the normal range in this study, thus might obtain the relevance of reducing airway oedema and improving the TDI^2,19^.

As expected, higher IPAP would lead to higher leakage; however, in contrast to other studies, no difference could be found between the two groups in our study. This observation probably was because a lower IPAP level had been used compared with other investigations^11,15^. Another reason might be that we closely monitored the usage of the ventilator at home and instructed or helped the patient to deal with the problem during the follow-up period if the leakage was too large. Similar to results reported by Dreher *et al*.^11^ and Duiverman *et al*.^10^, no significant between-group difference was noted in daytime P_a_CO_2_, but a positive tendency in favour of high-pressure NPPV was observed in our study. In addition, the mean values of daytime P_a_CO_2_ in the high-pressure group were in the normal range, while patients in the low-pressure group were still hypercapnic. Similar findings were seen in the HRQL, which was improved in both groups, but no significant difference could be detected. As for exercise tolerance, the percentage of the changes in the 6-minute walk distance got minimal clinically important difference^21,22^ with the values for high-pressure and low-pressure groups being 53.85% and 38.10%, respectively. Although we did not observe statistically significant differences between these two groups, probably because of the small sample size and the short follow-up period, our findings imply that high-pressure NPPV might be relevant to improve HRQL and exercise tolerance.

In this pilot study, the average IPAP level of high-pressure NPPV was only 21.15 cm H_2_O, and this pressure was lower compared with those in previous studies^10,11^. While owing to that the somatotype and upper airway resistance of Asians are quite different from those of westerners and with respect to the conventional IPAP level used in clinical practice^23^, the IPAP level of high-pressure NPPV in our study was a relatively high pressure. Furthermore, the back-up rate was not set higher than the spontaneous respiratory frequency to reach a controlled mode, which differed from prior studies. However, Murphy *et al*.^16^ pointed out that no additional benefit resulted from supplementing high IPAP with a high back-up rate, and they suggested that high pressure played a significant role in high-intensity NPPV.

Some limitations of this study should be mentioned. First, the power calculation for daytime PaCO_2_ was only 0.62 which was less than 0.75, indicated that larger sample size and longer follow-up time were needed in the further clinical trial. Second, we did not perform night-time arterial blood gas analysis; therefore, we did not obtain patient P_a_CO_2_ during nocturnal NPPV.

In this current pilot study, it implied us that the association between high-pressure NPPV and the improvement in TDI scores might exist when compared with low-pressure NPPV. In addition, a positive trend favouring high-pressure NPPV for improving P_a_CO_2_ and FEV_1_ was noticed. However, the sample size was not large enough and the follow-up period was quite not long. In the near future, we will proceed a longer follow-up time (more than a year) clinical study with large sample size determined by an accurate power calculation to determine what the best settings are for long-term NPPV for hypercapnic COPD patients. The inclusive patients will be randomly assigned to high-pressure group or low-pressure group using stratified block randomization, via a computer-generated block randomization sequence, with a block size of four.

For now, no unified method for setting up high-pressure NPPV has been established. Most of the trials utilized gradually increased IPAP depending on the patient’s tolerance^10–12^. However, from a respiratory physiology point of view, excessive IPAP may lead to lung hyperinflation, increased intrinsic positive end expiratory pressures, increased oxygen consumption, and ineffective work of breathing^17^. Therefore, seeking a method to establish individualized high-pressure NPPV is of vital importance.

In conclusion, high-pressure NPPV used for patients with chronic hypercapnic COPD is associated with improvement in TDI scores and a positive trend in favour of high-pressure NPPV for improving P_a_CO_2_ is observed. Use of an NIV equipped with built-in software allowed monitoring of the efficacy of home NPPV. A large sample size studies with greater follow-up period are necessary to determine the long-term effect of high-pressure NPPV and a more advanced method for establishing this new NPPV strategy is needed.

## Methods

The Ethics Committee of The First Affiliated Hospital of Guangzhou Medical University, Guangzhou, China approved this study, and all patients provided written informed consent before the study began. The trial was registered with ClinicalTrials.gov, number NCT02499718 (July 16, 2015). In this study, all methods were performed in accordance with the relevant guidelines and regulations.

In this pilot study, we investigated 34 patients recruited in a prior, prospective, multicentre, randomized, controlled trial^24^, using high-pressure (IPAP ≥ 20 cm H_2_O^9^) or low-pressure ventilation (IPAP ≤ 16 cm H_2_O^11^) for 3 months. Patients were clinically stable with chronic hypercapnic COPD with severe to very severe airflow limitation. Patients were judged to be clinically stable if they had no acute exacerbation^1,25^, which was characterized as an acute worsening of more than one respiratory symptom (new onset of or increase in sputum volume or purulence, wheezing, cough, dyspnoea, or fever) lasting for at least 2 consecutive days and did not have any changes in their conventional therapy for 1 month^3^. Exclusion criteria were: (1) other lung/pleural diseases (for example, bronchiectasis, bronchogenic carcinoma, neuromuscular disease) or thoracic deformity; (2) severe heart failure (New York Heart Association class IV), severe dysrhythmia, unstable angina, or malignant comorbidity; (3) obesity (BMI ≥ 35 kg/m²); (4) severe obstructive sleep apnoea syndrome; and (5) previous NPPV or any form of invasive ventilation.

All patients received regular, optimal pharmacologic treatments according to the GOLD guideline^1^. Patients were advised to use NIV at least 5 hours per day. They were recommended to use NPPV during sleep, but daytime usage was also acceptable. For this study, patients were provided with a Flexo ST 30 NIV ventilator (Curative Medical Technology Inc., Suzhou, People’s Republic of China) using a spontaneous/timed mode of ventilatory support. Expiratory positive airway pressure (EPAP) was set at a low level (4–6 cm H_2_O), and the IPAP was gradually increased according to the toleration of patient. The pressure support level (IPAP−EPAP difference) was more than 10 cm H_2_O^6^. The back-up rate was set at 16 breaths/min. The software built into the NIV could record parameters such as leakage, IPAP, EPAP, air flow, tidal volume, minute ventilation, and respiratory rate. Staffs monitored patients’ daily usage. If leakage was above 40 L/min, we guided the patients to tighten their head band or changed the mask with appropriate size. When patient’s daily usage below 5 hours, we reminded the patients to lengthen the usage time. Additionally, 24 h phone service was available for the patients in case of problems happened. Furthermore, if the problems could not be solved by phone, staff provided door-to-door service immediately. Also detailed data were extracted from the software and analysed every 4 weeks.

### Outcomes

The primary outcome was daytime P_a_CO_2_. Daytime arterial blood gas samples were taken with patients resting in a sitting position and breathing room air without having used NPPV for at least 1 hour^3^. Secondary outcomes were HRQL, based on the SRI Questionnaire^26^ and the CAT^27^; pulmonary function; 6MWD; blood oxygen saturation; and baseline/transition dyspnoea index^28^.

### Statistical analysis

The data analysis was conducted using IBM SPSS Statistics for Windows, Version 21.0 (IBM Corp., Armonk, NY, USA). 20 patients per group were needed in order to reveal a difference of 7 mmHg in daytime PaCO_2_ between high-pressure and low-pressure ventilation with an SD of 7 mmHg was assured from the previous study^13^. The study was designed to have a power of 80% and a two-sided significance level of 0.05. The data are presented as n (%), mean ± standard deviation (SD) or median ± range according to their distribution. *P*-values < 0.05 were considered statistically significant. For continuous variables, comparisons were performed using the independent samples t test for normally distributed data and a nonparametric test for data not normally distributed. For categorical variables, the percentages of patients in each group were compared using Pearson’s chi-squared test.

### Data Availability

All data generated or analyzed during this study are included in this published article.
