# Author Correction: High-pressure versus low-pressure home non-invasive positive pressure ventilation with built-in software in patients with stable hypercapnic COPD: a pilot study

**DOI:** 10.1038/s41598-018-22723-w

**Published:** 2018-03-22

**Authors:** Luqian Zhou, Lili Guan, Weiliang Wu, Xiaoying Li, Xin Chen, Bingpeng Guo, Yating Huo, Jiawen Xu, Yuqiong Yang, Rongchang Chen

**Affiliations:** 1 State Key Laboratory of Respiratory Disease, Guangzhou Institute of Respiratory Disease, the First Affiliated Hospital of Guangzhou Medical University, Guangzhou, China; 2 The First Affiliated Hospital/School of Clinical Medicine of Guangdong Pharmaceutical University, Guangzhou, China; 3 ZhuJiang Hospital of Southern Medical University, Guangzhou, China

Correction to: *Scientific Reports* 10.1038/s41598-017-17142-2, published online 01 December 2017

In the original version of this Article, Luqian Zhou, and not Rongchang Chen, was listed as the corresponding author. Correspondence and request for materials should be addressed to Rongchang Chen at chenrcstatekeylab@gmail.com.

In addition, the order of the author names was incorrectly given as ‘Rongchang Chen, Lili Guan, Weiliang Wu, Xiaoying Li, Xin Chen, Bingpeng Guo, Yating Huo, Jiawen Xu, Yuqiong Yang & Luqian Zhou’.

Finally,

“Rongchang Chen, Lili Guan, Weiliang Wu and Xiaoying Li contributed equally to this work.”

now reads:

“Luqian Zhou, Lili Guan, Weiliang Wu and Xiaoying Li contributed equally to this work.”

These errors have now been corrected in the HTML and PDF versions of this Article.

